# Diversity in tooth eruption and life history in humans: illustration from a Pygmy population

**DOI:** 10.1038/srep27405

**Published:** 2016-06-16

**Authors:** Fernando Ramirez Rozzi

**Affiliations:** 1 AMIS UMR 5288 CNRS - Université Paris V. Faculté de Chirurgie Dentaire, 1 rue Maurice Arnoux, 92120 Montrouge, France

## Abstract

Life history variables (LHV) in primates are closely correlated with the ages of tooth eruption, which are a useful proxy to predict growth and development in extant and extinct species. However, it is not known how tooth eruption ages interact with LHV in polymorphic species such as modern humans. African pygmies are at the one extreme in the range of human size variation. LHV in the Baka pygmies are similar to those in standard populations. We would therefore expect tooth eruption ages to be similar also. This mixed (longitudinal and cross-sectional) study of tooth eruption in Baka individuals of known age reveals that eruption in all tooth classes occurs earlier than in any other human population. Earlier tooth eruption can be related to the particular somatic growth in the Baka but cannot be correlated with LHV. The link between LHV and tooth eruption seems disrupted in *H. sapiens*, allowing adaptive variations in tooth eruption in response to different environmental constraints while maintaining the unique human life cycle.

The ultimate description of an organism is not just a description of its adult phase, but that of its life cycle^1^. Life history is defined as the allocation of an organism’s energy toward fitness processes such as growth, development, reproduction, and survival^2^,^3^. Life history variables (LHV) include age at weaning, age at sexual maturity, age at first breeding, interbirth interval and life span. LHV are closely correlated with ages of tooth eruption across primate species. Indeed, Smith^4^,^5^ has shown that age at the first molar eruption is highly correlated with age at weaning, age at female sexual maturity, interbirth interval, age at first breeding and, in male primates, age at sexual maturity (see ref. 6). Furthermore, the age when the first molar erupts is also highly correlated with the ages of eruption of all other tooth types and even with the total period of tooth eruption among primates^7^,^8^. The strong correlation between tooth development and LHV makes it possible to use tooth development, and especially age at molar eruption, as a good proxy for inferences about LHV in hominids (i.e. refs 4 and 5).

Harvey & Clutton-Brock^6^ observed that most measurements in species within genera and in genera within subfamilies tend to be very similar, and close correlations have been determined in high-ranking taxa (higher than subfamily) in primates. It is true that no major differences have been reported among populations in extant hominid species, except in modern humans, which display a wide range of morphological variation. The link between LHV and tooth eruption ages has not been addressed at the intraspecies level among populations of a polymorphic species such as *Homo sapiens*. It is therefore of interest to understand how tooth eruption ages relate to LHV in a population at the extreme end of the size range of modern humans.

African pygmies inhabit equatorial rain forest (see Supplementary Text) and have a characteristically short stature at the most extreme of human variation. The average stature of African pygmies is less than 1.55 metres^9^. The pygmy phenotype itself is usually interpreted as an adaptation to life in equatorial rain forests^10^. The causes driving the pygmy phenotype are not clear as yet. The correlation between the average height in pygmy populations and the degree of Bantu genetic introgression suggest that the pygmy phenotype is linked to genetic changes^11^; the identification of some SNPs associated with stature in their genome support this interpretation^12^,^13^. However, beyond this limited information on SNPs, the full spectrum of biological modifications in pygmies is unknown^14^. The analysis of few individuals suggests that some kind of deficiency in the GH-IGF axis is responsible for the short stature of pygmies^15^. Recently, based on the most extensive study on pygmies to date, we suggested that the pygmy phenotype in the Baka from south-eastern Cameroon results from changes in growth during earliest infancy, and therefore that the pygmy phenotype results from a low growth rate during the first 2 years of life, which produces a lasting delay in overall growth compared with standard ref. 16. Adult height (average for males = 153.5 cm, average for females = 146.7 cm) is attained at 20 and 18 years respectively. LHV in the Baka (median age at menarche age at first pregnancy, interbirth interval) do not differ from those in standard populations^16^.

Baka pygmy phenotype is principally due to a particular growth process during infancy which is the period when all permanent teeth (except M3) begin to form. This particular growth pattern could be accompanied by changes in dental development. The presence of a population of Baka pygmies of known age enabled us to address, for the first time, the maturation of the dentition in pygmies directly, with no prior assumption. Moreover, the growth pattern as well as life history variables are known for this population, so that we were able to assess tooth development in relation to body growth and development. The aim of our work is twofold: first, to characterize tooth development in Baka pygmies and establish the chronology and the sequence of tooth emergence; secondly, to discuss whether variations in tooth development match those observed in growth and in LHV. Tooth eruption ages are closely linked with LHV, and since these are similar in Baka and standard populations, one would expect tooth eruption to occur at similar ages as in other populations. If confirmed, this would strengthen the suggested correlation between tooth development and LHV, making the former a good proxy for the latter. If not, tooth development varies in ways that cannot be predicted by life history variables.

## Results

### Eruption

**Figure 1:**
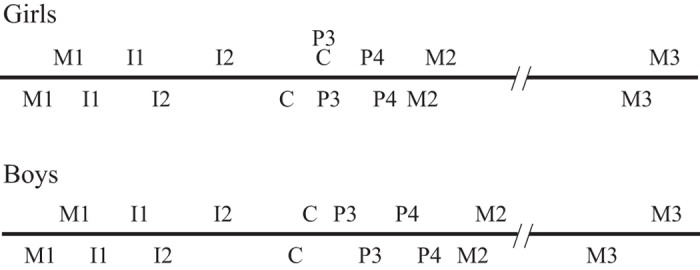
Sequence of tooth eruption in Baka pygmies.

Median tooth eruption ages among pygmies are given in Table 1. Eruption occurs earlier in females than in males for all tooth classes and much earlier for M3. In both sexes, the lower anterior teeth and molars erupt before the upper ones, but upper premolars erupt before the lower ones. Interestingly, the interval between the eruption of lower and upper teeth is longer in boys than in girls, except for canines and M1. The interval between the eruption of lower and upper lateral incisors is close to one year in both sexes. C and P3 eruptions occur close together in time, producing a difference in sequence between the sexes, with the upper P3 emerging before the canines in males but after the canines in females (Fig. 1).

**Figure 2:**
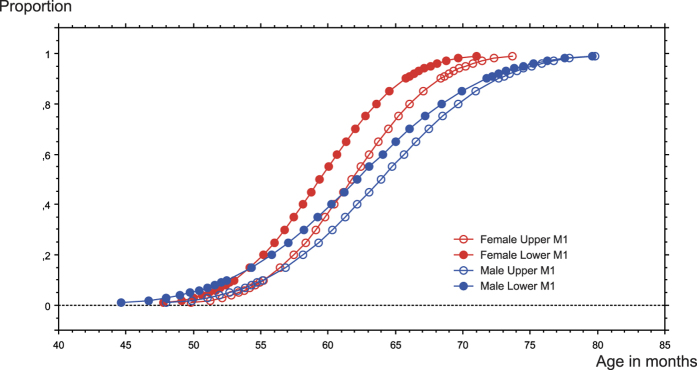
Distribution of M1 eruption in females (red) and males (blue). Lower M1 (symbols in block colours) erupt earlier than upper ones (blank symbols). Median eruption ages are earlier in females than in males, but the range is larger in males (close to two years in females and almost 3 years in males).

Probit analysis indicates that the earliest lower M1 eruption (probability 0.01) occurs at 3.98 years of age in females and 3.72 years in males (see Supplementary Table 1). The lower age in males was influenced by a single individual with the M1 in eruption at 45 month of age. The closest to this value was found a year later. If this single male is removed from the analysis, the median age of eruption increases by only ten days. All females have their lower M1 in eruption (probability 0.99) at 5.92 years of age; in males, this is observed at 6.63 years of age. The age range in males is greater than in females (Fig. 2).

Advanced M1 eruption was recorded in four males and two females. In all of them, other teeth erupted at an earlier age than median ages obtained for the population. Late eruption of M1 was observed in one male and one female; these individuals also showed delayed eruption in other tooth types.

### Full occlusion

Median ages at full occlusion are given in Table 2. Full occlusion is reached earlier in females than in males for all tooth classes. The lower anterior teeth and molars are fully occluded before the upper ones, except for M3. In males, full occlusion is observed first in the upper premolars whereas in females, lower P3 is ahead of upper P3 and lower and upper P4 reach full occlusion at the same time. The interval between full occlusion of the lower and upper teeth is shorter than their emergence (see Supplementary Table 2). While the duration of eruption is short for P4 and M1, eruption takes over a year for lower I2 in both sexes as well as for C and upper P3 in males.

The longitudinal data used here were obtained once a year for a minimum of four years from 64 individuals of 3 to 13 years of age. Because oral cavity inspections were carried out only on one day a year, there was a low probability of observing processes occurring within an interval shorter than one year, which is the case for M1 as these erupt in less than 6 months (see Supplementary Table 2). On the other hand, the duration of eruptions lasting more than one year, of I2 for example, is easily recorded through one annual inspection; in some individuals, we could even expect to record an emergence event lasting over two successive annual inspections. The longitudinal data shows that I2 emergence was recorded in 13 boys and 4 girls in two consecutive years and C emergence was observed in 8 boys during two successive annual inspections. These observations confirm the long duration of eruption for these tooth classes.

### Comparison

Dental development has been studied in many populations around the world, but this kind of study has rarely, if ever, been made in traditional societies such as hunter-gatherers or pastoralists, or in remote farming societies. A key aspect of dental development is the timing of eruption. This requires data on individual chronology, which is nearly always lacking for traditional societies. The lack of absolute chronology is sometimes resolved by using dental chronology to estimate individual age (i.e. ref. 17). However, this approach assumes that dental chronology based on tooth eruption ages is similar in all populations, but this is not the case. In an extensive review of published studies on different human populations, Liversidge^18^,^19^ provides a large amount of data on the eruption of permanent teeth and shows that the timing can vary among populations by as much as six months in median age. Although there is no pattern of difference in the average timing of tooth eruption among populations from different geographical areas, in some sub-Saharan African populations, but not all, some teeth erupt earlier than in populations inhabiting other regions of the world^18^.

**Figure 3:**
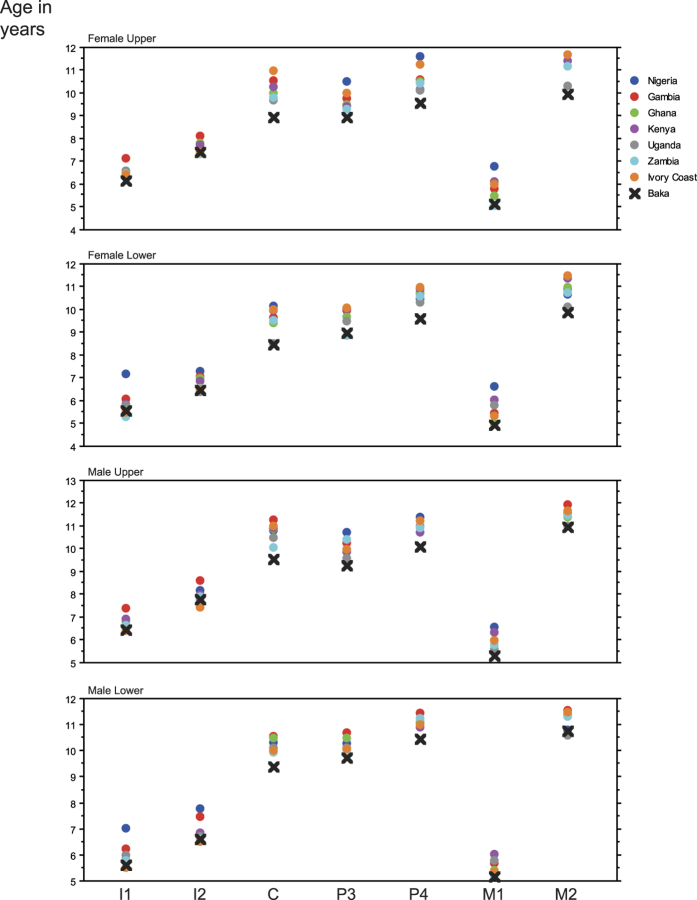
Tooth eruption ages in the Baka compared with other sub-Saharan groups. The Baka are always at the lower end, indicating more precocious tooth eruption. See Table 3.

The growth of dentition in the Baka pygmies is compared with data for other African populations in Table 3. In general, the Baka share many aspects with other populations: girls are ahead of boys in eruption timing, lower teeth precede upper teeth except for premolars, and the sequence of eruption between C and P3 varies between the sexes. In contrast, however, the Baka appear to be a unique group regarding ages of tooth eruption, with the majority of tooth classes erupting earlier than any other group (Fig. 3). Uniquely also, the large age difference for M2 eruption between Baka males and females (around 1 year) has not been recorded in any other population.

**Figure 4:**
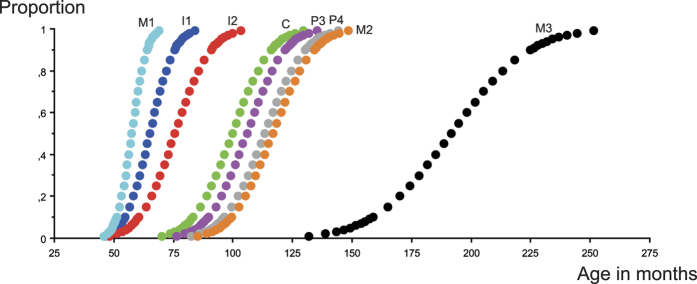
Range of eruption durations for lower teeth in girls. The range increases for teeth erupting at a later age.

While most studies on the timing of tooth eruption provide median or average ages with a standard deviation, they rarely give the full range of variation on the timing of eruption for different tooth classes. In the Baka, the smallest ranges are found for I1 and M1 in both sexes, while the largest corresponds to C and P4 in boys and M2 in girls (Suppl. Matter Table 1). The range is highly correlated with the timing of eruptions in both sexes (P < 0.01) (Fig. 4). Ranges for the Baka do not seem to differ from those reported for a Danish population, at nearly 2.5 years for M1 and 4 to 5.5 years for M2^20^, but are much greater than those obtained for a French population in which the largest range, 3 yrs and 8 months, was observed for upper P4 ref. 21.

Because the range of variation increases over time, it is to be expected that M1 will take the shortest time to reach the occlusal plane. However, we found a short eruption time for P4, and the longest time for lower I2 (see above). These exceptions have already been observed in other populations^20^. Cattell^22^ reported a long interval for the upper I2 (9.05 months) in European Americans and mentions a duration of 18 months for another tooth class but without specifying which one. In a cross-sectional study of Jordanians, the shortest duration of eruption was also found in M1, with lower values than in the Baka; the longest duration of eruption, close to one year, was for M2 ref. 23.

## Discussion

The life cycle of modern human is unique among primates, mainly for the long lifespan and growth spurts at adolescence associated with other specific life history variables such as relatively helpless newborns, early weaning, an extended period of offspring dependency, late onset of reproduction, relatively short interbirth intervals, unusual secondary sexual characteristics and menopause^24^. The close correlations between LHV and tooth eruption ages in primates^4^,^7^ are used as an approach to the study of life cycles in extinct hominids. However, some scholars have suggested that these correlations may be artefacts of statistical analysis, or that only one variable is closely linked to the life cycle and is in fact the causal factor of the apparent correlations between other variables (i.e. refs 25, 26, 27). Taking a more critical view, Bermudez de Castro *et al*.^28^ question whether the synchronicity of the delayed appearance of parameters in humans is simply an obvious result of a slowdown in different physiological processes. However the close correlation between LHV and tooth emergence ages in primates is difficult to interpret as random parallelism. It is true that LHV have not evolved at the same pace among primates and that although general tendencies can be detected, it seems they have evolved in a mosaic pattern^24^.

Unique among primates is the wide morphological variation observed in *H. sapiens*. Average adult stature differs widely among modern human populations and since adult stature is the final result of growth, this suggests that the underlying growth processes responsible for the diversity must be equally diverse. A longitudinal study conducted over 8 consecutive years of a large Baka population ranging in age from 0 to 25 years of age established that at birth pygmies are similar to standard populations but that a significant decrease in growth is observed from birth to 2 years of age, resulting in a smaller size by age 3^16^. From 3 years of age onwards, their growth follows a pattern close to that observed in the lower percentiles in other populations, with a clear growth spurt during adolescence. The final adult stature reached at the standard age maintains the gap already established during infancy. These growth data are a key to understanding how the pygmy phenotype is acquired^16^. Importantly, the Baka growth pattern conforms to the standard human life cycle, with a growth spurt at adolescence and adult size reached at around 20 years of age (the lifespan in pygmies is not known). Thus the LHV in the Baka do not differ from LHV in standard populations; changes in body size are therefore not accompanied by changes in LHV. One would therefore expect tooth eruption ages not to differ in the Baka either. However, Vallejo-Bolaños & España-López^29^ observed that in short individuals in a population, tooth development is delayed in relation to both chronological age and skeletal growth. Similar results are found in studies of people with growth disorders. People affected by growth hormone deficiency and Laron type dwarfism show a delay in tooth development which is even more pronounced than in skeletal growth in relation to chronological age (i.e. refs 30 and 31). Since growth in Baka pygmies is characterised by a particular pattern during infancy, any differences in the ages of tooth eruption are likely to concern only the teeth formed during infancy, e.g. the first molars.

Studies on tooth eruption ages based on counting developing teeth in the mouth have been carried out in many different groups and countries. They show that variations do exist among populations. Liversidge^18^ suggests that tooth eruption ages vary, although less so than the timing of the crown and root growth stages. She observed that in some African populations, some teeth erupt earlier than in others inhabiting different regions of the world. However, these differences in tooth eruption rarely exceed one standard deviation^18^.

The main distinction between the Baka and other populations concerns the ages of tooth eruption. In the Baka, all teeth erupt earlier than in any other African population (Fig. 3). Early eruption of permanent teeth in the Baka is likely to produce an adaptive advantage; it can be related to earlier weaning and a quicker shift to a solid food diet, which enables the mother to start a new pregnancy. The interbirth interval of 2–3 years in the Baka is close to the figures for developing countries^16^. However, the average interbirth interval proposed for forager societies is 41.3 months (3.5 years), varying from 37.1 to 45.1 months (see ref. 32). These intervals are much longer than those in the Baka. The difference between the Baka and other forager societies probably results from the fact that the average is obtained from models and not, as for the Baka, from real chronology. However, the possibility that precocious tooth eruption in the Baka is related to a distinctively short interbirth interval enabling faster reproduction, cannot be ruled out.

Growth in the Baka is characterised by a marked decrease of velocity during infancy, from birth to 2 years of age^16^. From an age-scale perspective, the rapid decrease in velocity during these two years can also be seen as a form of precocious development compared to standard populations, where growth slows down over three years. In other words, the period of development characterised by a significant decrease in velocity is shorter and thus ends earlier in the Baka than in other populations. The formation of the crown of anterior teeth and M1 takes place during this period and formation of premolars and M2 starts around 2.5 years of age. The earlier eruption of permanent teeth in the Baka is probably related to their precocious development from birth to 2 years of age. Pelsmaekers and colleagues^33^ have suggested that variation in dental age is explained by additive genetic influences but principally by environmental factors during the prenatal, natal and immediate post-natal periods. It should be noted that the low growth rate in the Baka that occurs only during infancy are accompanied by reduction in tooth eruption ages throughout the period of growth.

The median age of M2 eruption in the Baka pygmies is around 9.93 years in girls and around 10.86 years in boys (Table 1). These are much earlier ages than those reported for other sub-Saharan populations (Table 3). LHV in the Baka are similar to those in standard populations^16^. When age at M2 eruption is compared with age at menarche, we observe that the gap in sub-Saharan populations ranges from 0.89 to 3.85 years but it reaches 4.57 years in the Baka pygmies (Supplementary Table 3). The long period between M2 eruption and menarche confirms the precocity of eruption. Tooth development can therefore be characterised as precocious in regard to the life cycle of the Baka.

In the last 30 years, many studies have focused on growth and development in fossil hominids in order to understand when and how the life cycle of modern humans appeared during hominin evolution. The presence of incremental lines in dental tissues makes it possible to determine ages of tooth eruption in young individuals and, more broadly, to calibrate growth in fossil hominins against real chronology. Ages of molar emergence differ considerably between modern humans and chimpanzees, e.g. mandibular M1 emerges at 3.17 years (range = 2.14–3.99 yrs) in captive chimpanzees and at 6.3 years (range 5.2–7.4 yrs) in modern humans^34^. Based on the high correlation between tooth development and life history variables, it can be inferred that fossil hominins with ages of molar eruption closer to those of chimpanzees would most probably have had no growth spurt, an earlier age at menarche and a shorter lifespan than modern humans. In an extensive study on the growth of the Nariokotome boy, Dean and Smith^35^, based on the microanatomy of hard tissues, observed precocious tooth development with gingival emergence of M2 at a very early age, suggesting that this boy’s growth and development pattern was closer to that of an ape than a human. The result from this study agrees with the estimation of M2 eruption in *Homo erectus* at approximately 9 years of age, based on the correlation between M2 eruption and cranial capacity in primates^3^,^7^. Dean and Smith conclude that although the growth pattern of *H. erectus* is probably unique, it is more like that of chimpanzees. M2 eruption occurs earlier in the Baka than in any other population and closer to the estimated age for *H. erectus*. If the reasoning followed to infer the life history of the Nariokotome boy is applied to the Baka, the ages of molar eruption would suggest that the Baka life cycle is closer to that of *H. erectus* than to *H. sapiens*. However, we know that the LHV of the Baka are similar to those of standard populations and that the diagnostic life cycle of the Baka is that of *H. sapiens*. Therefore, estimations of LHV and life cycle in the Baka based on ages of tooth eruption would be erroneous. In other words, tooth eruption ages cannot predict the standard LHV presented by the Baka.

Baka tooth development thus appears to be precocious when compared with other populations. This is reinforced by the fact that early tooth eruption ages are accompanied by LHV similar to those of standard populations; tooth eruption ages follow a regular chronological pattern but seem to be independent from the life cycle. This observation does not invalidate the correlations established in comparisons of high-ranking primate taxa, but does suggest that intraspecies variation can modify patterns observed in high-ranking taxa to adapt to particular environmental conditions. Body size variations reveal variations in growth that are independent of LHV^16^. These cannot be considered solely as adaptations to distinct environmental conditions, because they would reflect, more importantly, a capacity to transform growth in response to constraints, thus enabling different kinds of adaptation. One such adaptation in *H. sapiens* could be a dissociation between LHV and tooth development allowing body growth and tooth development to adapt in different directions while maintaining similar life stages.

## Methods

In the Moange-le-Bosquet locality in south-eastern Cameroon, nuns with medical training had kept records of Baka pygmy births from the 1970 s. The birth records from the 1970 s and early 1980 s have been lost, but those from 1988 onward were available for our study. From 2007 to 2013 (7 years), we travelled to Bosquet and followed individual growth in dentition on a yearly basis. The work to be carried out was explained to the Baka people. Because Baka are illiterate, we obtained the informed oral consent of the head of the village and the informed oral consent from all the individuals. Our work was carried out in accordance with the approved guidelines, as part of the international agreement between, and with the approval from, the IRD and the Ministry of Scientific Research and Technology of Cameroon.

Oral observations were made in all the Baka individuals who came to us but only those whose birth date had been recorded by the nuns and who had two Baka parents were included in the study. About 550 individuals were thus included, ranging from 3 to 20 years of age (Table 4). The dataset was built up with mixed longitudinal and cross-sectional data. This population had never received any oral health care. In order to establish the chronology and the sequence of tooth eruption, dentition growth was measured by characterising the stage reached by each tooth in the jaws through inspecting the oral cavity. For each tooth, three events were recorded: absence, in eruption and full occlusion. “Absence” means that a given tooth was not present in the oral cavity, and also refers to permanent teeth when temporary teeth were in place. “In eruption” means that any part of the tooth was visible or had penetrated the oral mucosa but had not yet reached the occlusal plane. “Full occlusion” means that the tooth had reached the occlusal plane. The age of each individual was recorded in days and divided by 365 to obtain age in years. Individuals were grouped by sex into 6-month age classes. The age given corresponds to the mid-point of the class (age +/−3 months). Through probit regression (SPSS), the median age at eruption and the median age at full occlusion (95% confidence limits and standard error) were calculated for each tooth. In this respect, the result for “in eruption” and “full occlusion” was binary: “absence” *vs*. “in eruption-full occlusion” for eruption and “absence-in eruption” *vs.* “full occlusion” for full occlusion. The duration of tooth eruption was obtained for each tooth class and corresponds to the interval in time between the median of two events (median for “full occlusion” – median for “in eruption”)^23^. We are aware that this value is approximate as we did not record the actual moment when a tooth emerged or the final period of eruption just before a tooth reached the occlusal plane. However, the data can be usefully compared with the few studies that have obtained durations of eruption in similar or less precise ways^20^,^22^,^23^.

The number of individuals included in the sample to calculate median ages is provided in Table 4. The difference in sample size among tooth classes results from the fact that only individuals in the age classes actually included in the probit analysis are counted. For instance, the age classes used for probit analysis extend from the last age class with 0% occurrence of a given tooth event to the first age class with 100% of occurrence of the same tooth event. In our study, there is 0% full occlusion of the upper I1 at the 63 month age class (+/−3 months) and 100% in the 117 month age class (+/−3 m), while there is 0% occurrence of full occlusion of the lower I1 in the 57-month age class and 100% in the 117-month age class; the sample size for the upper I1 is smaller than for the lower I1 (283 *vs*. 313) because observations of the 57-month age class are not included in the analysis for the upper I1.

Probit analysis enabled us to analyse longitudinal data as if it were cross sectional data. Longitudinal data was used to assess the duration of tooth eruptions obtained by the difference between the median ages of two events. For the longitudinal analysis, only individuals observed for at least four years were kept, i.e. 64 individuals (girls = 25; boys = 39) ranging from 3 to 13 years of age, an interval that covers the eruption of all permanent teeth except M3. Longitudinal data was also used to check whether individuals with precocious or late eruption of M1 also showed early or late eruption in other tooth classes. Only individuals studied for at least four years were included in this analysis. The individuals studied were those where M1 eruption occurred 0.25 years earlier or later than the median ages obtained for the population.

## Additional Information

**How to cite this article**: Ramirez Rozzi, F. Diversity in tooth eruption and life history in humans: illustration from a Pygmy population. *Sci. Rep.* **6**, 27405; doi: 10.1038/srep27405 (2016).

## Supplementary Material

Supplementary Information

## Figures and Tables

**Table 1:** Median tooth eruption ages in the Baka (*95*% *CI*) (*SE*).

	**Female**		**Male**	
	**Lower**	**Upper**	**Lower**	**Upper**
I1	**5**.**58**	**6**.**14**	**5**.**62**	**6**.**47**
	(4.39–6.8)	(5.95–6.33)	(5.36–5.86)	(6.12–6.79)
	0.015	0.012	0.018	0.01
I2	**6**.**46**	**7**.**43**	**6**.**62**	**7**.**77**
	(6.0–6.9)	(7.19–7.67)	(6.38–6.97)	(7.35–8.19)
	0.008	0.006	0.007	0.007
C	**8**.**46**	**8**.**93**	**9**.**4**	**9**.**55**
	(8.24–8.69)	(8.7–9.16)	(8.78–10.02)	(9.04–10.07)
	0.008	0.007	0.006	0.006
P3	**8**.**97**	**8**.**94**	**9**.**73**	**9**.**27**
	(8.59–9.35)	(8.71–9.16)	(9.50–9.97)	(9.04–9.51)
	0.009	0.008	0.007	0.006
P4	**9**.**6**	**9**.**57**	**10**.**47**	**10**.**09**
	(9.37–9.84)	(9.34–9.81)	(10.23–10.72)	(9.83–10.35)
	0.007	0.007	0.006	0.005
M1	**4**.**95**	**5**.**15**	**5**.**18**	**5**.**33**
	(4.81–5.09)	(5.01–5.28)	(5.01–5.34)	(5.17–5.48)
	0.029	0.029	0.015	0.018
M2	**9**.**88**	**9**.**98**	**10**.**76**	**10**.**97**
	(9.49–10.23)	(9.48–10.41)	(10.52–11)	(10.72–11.22)
	0.008	0.007	0.008	0.007
M3	**16**.**13**	**16**.**66**	**18**.**33**	**19**.**63**
	(15.52–16.94)	(16.1–17.43)	(17.61–19.46)	(18.60–21.74)
	0.004	0.042	0.006	0.006

*If age classes are built every twelve month values are not different, 18.16 years for lower M3 and 19.19 years for upper M3 in males.

**Table 2:** Median ages at full occlusion in the Baka (*95% CI*) (*SE*).

	**Female**		**Male**	
	**Lower**	**Upper**	**Lower**	**Upper**
I1	**6**.**37**	**6**.**88**	**6**.**46**	**7**.**12**
	(5.94–6.79)	(6.45–7.26)	(5.73–7.09)	(6.92–7.31)
	0.011	0.011	0.01	0.009
I2	**7**.**69**	**7**.**98**	**8**.**31**	**8**.**74**
	(7.46–7.91)	(7.74–8.21)	(7.94–8.69)	(8.41–9.11)
	0.008	0.007	0.005	0.006
C	**9**.**35**	**9**.**7**	**10**.**55**	**10**.**58**
	(9.0–9.69)	(9.46–9.93)	(10.16–10.92)	(10.31–10.85)
	0.008	0.007	0.006	0.006
P3	**9**.**61**	**9**.**7**	**10**.**5**	**10**.**44**
	(9.38–9.84)	(9.47–9.91)	(10.26–10.74)	(10.22–10.68)
	0.007	0.009	0.008	0.008
P4	**9**.**87**	**9**.**87**	**10**.**91**	**10**.**71**
	(9.4–10.28)	(9.63–10.11)	(10.66–11.16)	(10.46–10.96)
	0.007	0.008	0.007	0.006
M1	**5**.**25**	**5**.**35**	**5**.**51**	**5**.**56**
	(5.13–5.39)	(5.19–5.52)	(5.33–5.67)	(5.38–5.72)
	0.033	0.018	0.014	0.015
M2	**10**.**29**	**10**.**37**	**11**.**39**	**11**.**58**
	(10.02–10.55)	(10.1–10.63)	(10.97–11.82)	(11.31–11.87)
	0.006	0.006	0.007	0.006
M3	**17**.**01**	**16**.**77**	**21**.**47**	**20**.**67**
	(16.52–17.68)	(16.29–17.39)	(19.5–33.5)	(19.16–24.94)
	0.006	0.006	0.02	0.006

**Table 3:** Comparison with tooth eruption ages in other African populations.

**Sex**			**I1**	**I2**	**C**	**P3**	**P4**	**M1**	**M2**	**M3**
F	Nigeria^36^,^37^	upper	6.44	7.52	10.27	10.51	11.62	6.77	11.16	
F	Gambia^38^	upper	7.13	8.10	10.56	9.78	10.60	5.80	11.19	
F	Ghana^39^	upper	6.50	7.80	10.00	9.50	10.50	5.50	11.40	
F	Kenya^40^,^41^	upper	6.55	7.71	10.26	9.40	10.15	6.13	11.40	18.48
F	Uganda^42^	upper	6.60	7.40	9.70	9.30	10.10	6.00	10.30	
F	Zambia^43^	upper	6.47	7.32	9.81	9.30	10.45	5.06	11.18	
F	Côte d’Ivoire^44^	upper	6.42	7.42	11.00	10.00	11.25	6.00	11.67	
F	Baka	upper	**6**.**14**	**7**.**43**	**8**.**93**	**8**.**94**	**9**.**57**	**5**.**15**	**9**.**98**	**16**.**66**
F	Nigeria^36^,^37^	lower	7.19	7.31	10.16	10	10.46	6.63	10.68	
F	Gambia^38^	lower	6.08	7.07	9.64	9.96	10.69	5.46	10.91	
F	Ghana^39^	lower	5.60	7.00	9.40	9.70	10.80	5.00	11.00	
F	Kenya^40^,^41^	lower	5.83	6.86	9.96	10.05	10.90	6.03	11.39	17.73
F	Uganda^42^	lower	5.80	6.40	8.50	9.50	10.30	5.80	10.10	
F	Zambia^43^	lower	5.31	6.55	9.51	8.87	10.59	5.35	10.74	
F	Côte d’Ivoire^44^	lower	5.50	6.50	10.00	10.08	11.00	5.33	11.50	
F	Baka	lower	**5**.**58**	**6**.**46**	**8**.**46**	**8**.**97**	**9**.**60**	**4**.**95**	**9**.**88**	**16**.**13**
M	Nigeria^36^,^37^	upper	6.87	8.19	10.82	10.74	11.38	6.55	11.65	
M	Gambia^38^	upper	7.38	8.60	11.29	10.26	11.23	6.00	11.96	
M	Ghana^39^	upper	6.80	8,00	10.90	10.00	11.00	5.50	11.40	
M	Kenya^40^,^41^	upper	6.91	7.99	10.93	9.87	10.74	6.32	11.54	18.83
M	Uganda^42^	upper	6.60	7.80	10.50	9.60	11.00	5.60	11.00	
M	Zambia^43^	upper	6.63	7.89	10.06	10.42	10.94	5.77	11.46	
M	Côte d’Ivoire^44^	upper	6.42	7.42	11.00	10.00	11.25	6.00	11.67	
M	Baka	upper	**6**.**47**	**7**.**77**	**9**.**55**	**9**.**27**	**10**.**09**	**5**.**33**	**10**.**97**	**19**.**63**
M	Nigeria^36^,^37^	lower	7.03	7.77	10.31	10.29	11.08		10.81	
M	Gambia^38^	lower	6.22	7.46	10.55	10.70	11.44	5.67	11.56	
M	Ghana^39^	lower	5.80	6.60	10.50	10.50	11.10	5.40	11.30	
M	Kenya^40^,^41^	lower	5.83	6.86	9.96	10.05	10.90	6.03	11.39	18.10
M	Uganda^42^	lower	6,00	6.70	10.10	10.10	11.00	5.80	10.60	
M	Zambia^43^	lower	5.79	6.62	9.95	9.90	11.23	5.19	11.30	
M	Côte d’Ivoire^44^	lower	5.50	6.50	10.00	10.08	11.00	5.33	11.50	
M	Baka	lower	**5**.**62**	**6**.**62**	**9**.**40**	**9**.**73**	**10**.**47**	**5**.**18**	**10**.**76**	**18**.**33**

For Côte d’Ivoire, females and males are counted together. It is worth to note that results from Zambia are extrapolations and that mean values proposed by Gillett are lower than the observed data range (Tables 1 and 2: 450)^43^. See ref. 18.

**Table 4:** Sample size.

		**Females**		**Males**	
		**Lower**	**Upper**	**Lower**	**Upper**
In eruption	I1	227	227	312	287
	I2	296	288	364	275
	C	255	276	330	330
	P3	216	276	296	352
	P4	265	285	330	330
	M1	134	127	247	195
	M2	232	274	242	242
	M3	263	213	145	130
Full occlusion	I1	277	243	313	283
	I2	243	284	313	313
	C	288	288	279	266
	P3	288	257	208	267
	P4	274	257	266	301
	M1	128	179	230	195
	M2	274	274	242	255
	M3	166	166	98	130
