# Supplementary material for: Diversity in tooth eruption and life history in humans: illustration from a Pygmy population
Source: Sci Rep. 2016 Jun 16;6:27405. doi: 10.1038/srep27405 (PMC4910065; doi:10.1038/srep27405)
Supplement: Supplementary Information [file srep27405-s1.pdf]

Diversity in tooth eruption and life history in humans: illustration from a Pygmy population

Short title: **Tooth eruption in a Pygmy population**

Author affiliation: AMIS UMR 5288 CNRS - Université Paris V. Faculté de Chirurgie Dentaire, 1 rue Maurice Arnoux, 92120 Montrouge, France.

Fernando Ramirez Rozzi, Faculté de Chirurgie Dentaire, 1 rue Maurice Arnoux, 92120 Montrouge, France. +33 664006179. ramrozzi@yahoo.fr, fernando.ramirez-rozzi@cnrs.fr

Keywords: Baka pygmies, dental development, life history variables, growth

Supplementary Table 1: Probit analysis limits, first record and last record for "in eruption" in years

	Female			Male		
	first	last	range	first	last	range
LI1	4.00	7.15	3.16	4.30	6.94	2.64
LI2	4.17	8.75	4.59	3.97	9.27	5.30
LC	5.99	10.94	4.95	6.33	12.47	6.15
LP3	6.52	11.42	4.90	6.96	12.50	5.54
LP4	7.03	12.18	5.16	7.67	13.27	5.59
LM1	3.98	5.92	1.93	3.72	6.63	2.91
LM2	7.25	12.51	5.26	8.20	13.33	5.13
UI1	4.36	7.93	3.57	4.49	8.45	3.95
UI2	4.53	10.33	5.80	4.93	10.62	5.69
UC	6.27	11.59	5.32	6.57	12.53	5.96
UP3	6.42	11.45	5.03	6.33	12.22	5.89
UP4	6.94	12.20	5.26	6.93	13.25	6.32
UM1	4.15	6.14	1.99	4.00	6.65	2.65
UM2	7.19	12.77	5.58	8.19	13.74	5.55

The ‘first’ column indicates the age at which 1% of individuals are at this developmental stage (probit = 0.01); the ‘last’ column indicates the age at which 99% of individuals have reached this developmental stage (probit = 0.99)

Supplementary Table 2: Duration of eruption*

	**Female**		**Male**	
	Lower	Upper	Lower	Upper
I1	**0.79**	**0.74**	**0.85**	**0.65**
I2	**1.23**	**0.55**	**1.69**	**0.97**
C	**0.89**	**0.77**	**1.15**	**1.03**
P3	**0.64**	**0.76**	**0.77**	**1.17**
P4	**0.27**	**0.3**	**0.44**	**0.62**
M1	**0.3**	**0.2**	**0.33**	**0.23**
M2	**0.41**	**0.39**	**0.63**	**0.62**
M3	**0.88**	**0.11**	**3.14**[§]	**1.04**

* obtained by subtracting the median ages of eruption from the median ages of full occlusion.

[§] Full eruption in M3 is not easy to assess and probably the small sample size in male influences this result.

Supplementary Table 3: Interval between age at M2 eruption and age at menarche

	Age of M2 eruption*	Age of menarche	Period
Nigeria	10.92	13.02[6]	2.1
Gambia	11.05	14.90[6]	3.85
Ghana	11.2	12.09[6]	0.89
Kenya	11.395	12.50[6]	1.105
Uganda	10.2	13.45[7]	3.25
Zambia	10.96	14.20[8]	3.24
Baka	9.93	14.5	4.57

* average age for upper and lower M2, see table 3.

Supplementary Text

Differences between African pygmies and non-pygmies also concern cultural and behaviour patterns as well as genetic distances. Pygmies have an economy based on hunting and gathering and a complex socio-economic relationship with their farming neighbours. Moreover, they are identified as pygmies by their culture and behaviour by farming neighbours, who in turn identify themselves as non-pygmies and are recognised as non-pygmies by pygmies, as well as by other non-pygmies (1, 2, 3). Pygmy populations are distributed across equatorial Africa in two main clusters. One is in East Africa (Rwanda, Uganda and eastern DRC) and comprises the Aka, Sua, Efe (also frequently called 'Mbuti') groups and the Batwa. The other cluster, in West Africa (Cameroon, Central Africa Republic, Congo, Gabon and western DRC), includes the Kola, Bongo, Koya, Aka, Baka and Twa. Pygmies share a common ancestor and split from Bantu-speaking populations at around 60,000 yrs BP (4): the split into an eastern and a western cluster would have taken place later than 20,000 yrs BP (4). Substantial admixtures between pygmies and non-pygmies have occurred in the last ~1000 years (5). Even today, the main barriers to admixture are the cultural and behavioural differences between these two groups (4).

Supplementary references

1 Bahuchet S. *La rencontre des agriculteurs. Les Pygmées parmi les peuples d'Afrique centrale*. Paris, Peeters-SELAF (1993).

2 Le Bomin S, Mbot J E. Sur les traces de l'histoire des Pygmées du Gabon : résultats de cinq ans de prospection. *J des Afr* 82: 277-318(2012).

3 Robillard M, Bahuchet S. Les Pygmées et les autres : terminologie, catégorisation et politique. *J des Afric* 82: 15-51(2012).

4 Patin E et al. Inferring the demographic history of African Farmers and Pygmy Hunter–Gatherers Using a MultilocusResequencing Data Set. *PLoS Genet* 5, e1000448. doi:10.1371/journal.pgen.1000448 (2009).

5 Patin E et al. The impact of agricultural emergence on the genetic history of African rainforest hunter-gatherers and agriculturalists. *Nat Commun* **5** (3163) doi: 10.1038/ncomms4163 (2014).

6 Hermanussen M et al. Adolescent growth: genes, hormones and the peer group. Proceedings of the 20th Aschauer soiree. *Pediatr Endocrinol Rev* 11: 336–349 (2014).

7 Odongkara Mpora B et al. Age at menarche in relation to nutritional status and critical life events among rural and urban secondary school girls in post-conflict northern Uganda. *BMC Womens Health* 14: 66. doi: 10.1186/1472-6874-14-66 (2014).

8 Pillai V K (1995) Age at menarche among adolescent females in Zambia: implication for family formation. *Int J Sociol Fam* 25: 33-38
